# Buprenorphine Exposure Alters the Development and Migration of Interneurons in the Cortex

**DOI:** 10.3389/fnmol.2022.889922

**Published:** 2022-05-04

**Authors:** Vanesa Nieto-Estévez, Jennifer J. Donegan, Courtney L. McMahon, Hannah B. Reiley, Teresa A. Chavera, Parul Varma, Kelly A. Berg, Daniel J. Lodge, Jenny Hsieh

**Affiliations:** ^1^Department of Neuroscience, Developmental and Regenerative Biology, The University of Texas at San Antonio, San Antonio, TX, United States; ^2^Brain Health Consortium, The University of Texas at San Antonio, San Antonio, TX, United States; ^3^Department of Pharmacology and Center for Biomedical Neuroscience, The University of Texas Health Science Center, San Antonio, TX, United States; ^4^Department of Psychiatry and Behavioral Sciences, Dell Medical School, The University of Texas at Austin, Austin, TX, United States; ^5^Audie L. Murphy Division, South Texas Veterans Health Care System, San Antonio, TX, United States

**Keywords:** buprenorphine, cortical development, opioid misuse disorder, interneurons, pregnancy

## Abstract

The misuse of opioids has reached epidemic proportions over the last decade, with over 2.1 million people in the United States suffering from substance use disorders related to prescription opioid pain relievers. This increase in opioid misuse affects all demographics of society, including women of child-bearing age, which has led to a rise in opioid use during pregnancy. Opioid use during pregnancy has been associated with increased risk of obstetric complications and adverse neonatal outcomes, including neonatal abstinence syndrome. Currently, opioid use disorder in pregnant women is treated with long-acting opioid agonists, including buprenorphine. Although buprenorphine reduces illicit opioid use during pregnancy and improves infant outcomes at birth, few long-term studies of the neurodevelopmental consequences have been conducted. The goal of the current experiments was to examine the effects of buprenorphine on the development of the cortex using fetal brain tissue, 3D brain cultures, and rodent models. First, we demonstrated that we can grow cortical and subpallial spheroids, which model the cellular diversity, connectivity, and activity of the developing human brain. Next, we show that cells in the developing human cortex express the nociceptin opioid (NOP) receptor and that buprenorphine can signal through this receptor in cortical spheroids. Using subpallial spheroids to grow inhibitory interneurons, we show that buprenorphine can alter interneuron development and migration into the cortex. Finally, using a rodent model of prenatal buprenorphine exposure, we demonstrate that alterations in interneuron distribution can persist into adulthood. Together, these results suggest that more research is needed into the long-lasting consequences of buprenorphine exposure on the developing human brain.

## Introduction

The Opioid Epidemic has reached epic proportions, with over 2.1 million people in the US suffering from opioid use disorder (OUD) (Chang et al., 2018). This epidemic affects all demographics of society, including women of childbearing age. In a 2017 study, 6.5 percent, or 21,000, pregnant women, reported illicit use of prescription opioids (Patrick et al., 2015; Ross et al., 2015), which has been associated with obstetric complications, such as preeclampsia and premature labor (Bashore et al., 1981; Hulse et al., 1998; Kaltenbach et al., 1998), and adverse neonatal outcomes, including neonatal abstinence syndrome, an array of symptoms caused by dysregulation of central, autonomic, and gastrointestinal systems (Kandall et al., 1976; Binder and Vavrinková, 2008; Hunt et al., 2008). To prevent these negative outcomes, the American Academy of Pediatrics and American College of Obstetricians and Gynecologists (ACOG) recommends opioid maintenance therapy to treat opioid use disorder during pregnancy. Buprenorphine, a partial μ- and κ-opioid receptor agonist, is used for outpatient opioid maintenance therapy, and is the first-line treatment for OUD in pregnant women recommended by ACOG (ACOG Committee on Health Care for Underserved Women and American Society of Addiction Medicine, 2012). Buprenorphine has been shown to be effective in reducing illicit opioid use in pregnant women (Johnson et al., 2003) and improves infant outcomes at birth compared to other opioid maintenance therapies, including methadone (Welle-Strand et al., 2013). However, buprenorphine can cross the placental barrier and directly affect the developing neonate (Nanovskaya et al., 2002). While some studies have found that *in utero* buprenorphine exposure does not affect early childhood growth, cognitive development, language abilities, or sensory processing (Tobon et al., 2019), others indicate that buprenorphine may have adverse effects on the developing fetus, including increased risk of prematurity and congenital malformations (Tobon et al., 2019). Further, behavioral consequences have been observed in preschool-aged children, including motor, memory, and attention deficits, along with hyperactivity and impulsivity (Sundelin Wahlsten and Sarman, 2013; Tobon et al., 2019). These conflicting results suggest that more research into the neurodevelopmental consequences of *in utero* buprenorphine exposure are urgently needed.

Studying fetal brain development in humans is extremely difficult due to ethical, time, and cost limitations. Further, multiple confounding factors, including obstetric complications and postnatal maternal care can have a major impact on the results. Therefore, in the current experiments, we use human pluripotent stem cells (hPSCs) to generate 3D brain spheroids, which retain more of the structural and functional properties of the developing human brain (Lancaster et al., 2013; PaŞca et al., 2015; Birey et al., 2017). The human cortex supports a variety of cognitive processes, including attention, memory, and consciousness. Cortical network activity is regulated by a balance of the activity of excitatory pyramidal cells and inhibitory interneurons. Alterations in this excitation/inhibition balance has been associated with a variety of neurodevelopmental and psychiatric disorders, including schizophrenia, autism, and intellectual disability (Marin, 2012). To generate excitatory neurons of the cortex, we grew cortical spheroids (hCS), which have ventricle-like structures surrounded by neuronal layers characteristic of the developing human cortex (PaŞca et al., 2015). Cortical interneurons are actually derived from a group of subpallial brain regions, the ganglionic eminences, before migrating tangentially into the cortex (Wonders and Anderson, 2005); therefore, to generate inhibitory interneurons, we grew subpallial spheroids (hSS), which express markers of developing interneurons (Bagley et al., 2017; Birey et al., 2017). These hCS and hSS were fused to examine the effect of buprenorphine exposure on cortical network development. Further, a rodent model was also used to confirm our *in vitro* findings and determine the long-term consequences of prenatal buprenorphine exposure.

In the current studies, we demonstrate that we can generate hCS and hSS, which resemble the developing human cortex and subpallium. We then show that in hCS, buprenorphine signals through the nociceptin opioid peptide (NOP) receptor, which is also expressed throughout the human fetal cortex. We also found that buprenorphine can alter markers of interneuron progenitors in the hSS. When we fused the two spheroid sub-types, buprenorphine altered interneuron migration into the hCS and increased network activity. In our rodent model of prenatal opioid exposure, we observed changes in interneuron distribution throughout the cortex after buprenorphine exposure, suggestive of long-lasting alterations in interneuron migration. Together, these results suggest that buprenorphine exposure can alter cortical development and produce persistent changes in the cortical network.

## Materials and Methods

### Pluripotent Stem Cell Generation and Maintenance

Human induced pluripotent stem cells (hiPSCs) were generated from a healthy 35-year-old male subject using an episomal reprogramming kit (Invitrogen). Approval for the study was obtained from the University of Texas at San Antonio IRB panel and informed consent was obtained from the subject. H9 ESCs were purchased from Wicell. Both hiPSCs and ESCs showed pluripotency markers and normal karyotype throughout the study (Supplementary Figures 1A,B) and were grown on matrigel-coated plates in mTeSR medium (Stem Cell Technology) containing the Rock inhibitor, Y27632 (10 μM, Selleck). In order to fluorescently label cells for migration analysis, the Neon Transfection Kit (Invitrogen) was used to transfect cells with CRISPR-mCherry (mCh) plasmids. Cells were dissociated using accutase, then 1 × 10^6 cells were resuspended in Buffer R containing 1 μg of CRISPR plasmid (AAVS1-T2 CRISPR in pX330) and 3 μg mCh plasmid (AAVS1-Pur-CAG-mCherry). Transfections were performed at 1,100 V, 20 msec, 2 pulses and cells were immediately re-plated in mTeSR medium containing Y27632. Three days after transfection, 0.3 μg/ml puromycin was added to the media for 5 days to kill non-transfected cells.

### Drugs

Buprenorphine was purchased from Cayman Chemical Company. In all experiments, organoids were treated with 2 ng/ml buprenorphine to approximate concentrations observed in human placental tissue (Concheiro et al., 2010). Oxycodone was purchased from Spectrum Chemicals. To compare an abused opioid known to affect neurodevelopmental (Larson et al., 2019), 20 ng/ml oxycodone was used (Kokki et al., 2012). For chronic treatment, drugs were added at day 50 for 10 days, period of active progenitor proliferation and migration.

### Spheroid Cell Culture

To grow hCS and hSS we used the method described by Pasca and colleagues with light modifications (PaŞca et al., 2015; Birey et al., 2017). Briefly, 9,000 stem cells were resuspended in 150 μl neural induction medium (DMEM/F12, 20% knock-out serum replacement, Glutamax, MEM-NEAA, 0.1 mM 2-mercaptoethanol, peni/strep) containing Y27632 (20 μM). Media was changed daily. On days 1-5, the SMAD inhibitors, dorsomorphin (5 μM, Sigma) and SB-431542 (10 μM, Tocris) were added to inhibit both the BMP and TGF-β signaling pathways. On days 6-24, media was replaced with neural medium (Neurobasal A, B-27 without Vitamin A, Glutamax, Peni/Strep) containing the growth factors, bFGF (20 ng/ml, Peprotech) and EGF (20 ng/ml, Peprotech). On day 25, spheroids were transferred to neural medium containing BDNF (20 ng/ml, Peprotech) and NT-3 (20 ng/ml, Peprotech) until day 42. hSS also received the Wnt pathway inhibitor, IWP-2 (5 μM, Selleckchem) on days 4-22 and the Smo pathway activator, SAG (100 nM, Selleckchem) on days 12-22 (Supplementary Figure 2A).

### RNA Sequencing

On day 60, hCS and hSS spheroids were homogenized and RNA was prepared using the Qiagen RNEasy Plus Micro Kit according to manufacturer’s instructions. Library construction, sequencing, and analysis were performed by NovoGene.

### Spheroid Immunohistochemistry

Spheroids were rinsed in DPBS, then fixed in 4% paraformaldehyde for 30 minutes - overnight at 4°C. After rinsing in DPBS, spheroids were incubated in 30% sucrose for 24-48 h at 4°C. Spheroids were embedded in OCT compound and frozen. A cryostat was used to cut 14-25 micron sections, which were mounted on gelatin-coated slides. For immunohistochemistry, slides were washed in PBS containing 0.3% Triton X-100, then blocked with 3-5% normal goat serum for 1 h. Slides were incubated in primary antibody (Table 1) overnight at 4°C in a humidified chamber. After washing, slides were incubated in secondary antibody (Jackson Immunoresearch, 1:500) for 1-2 h at room temperature. Slides were washed, DAPI (Sigma) was added to label nucleus, then coverslipped using Poly-Vinyl Alcohol (Sigma). Sections were imaged using a Leica Microscope (Spe8Info) and fluorescence intensity was analyzed using Image J software.

**TABLE 1:** Antibodies used for immunohistochemistry.

Antibody	Host Specie	Company	Cat. Number	Dilution
Anti-AC3	Rabbit	Cell Signaling	9661	1:400
Anti-ARX	Rabbit	Gift from Drs Morohashi and Kitamura		1:500
Anti-GFP	Chicken	AvesLab	GFP-120	1:500
Anti-Ki67	Rat	Accurate	OBT0030	1:250
Anti-Lin28	Rabbit	Cell Signaling	3978	1:1000
Anti-Nanog	Mouse	Thermoscientific	MA1-017	1:500
Anti-NKX2-1	Rabbit	Abcam	ab133737	1:500
Anti-NOP	Rabbit	Abcam	230477	1:100
Anti-Pax6	Rabbit	Biolegend	901301	1:300
Anti-Sox2	Rabbit	Millipore	AB5603	1:1000
Anti-Tuj	Mouse	Sigma	T8660	1:400

### qPCR

Human brain development, we first generated cortical spheroids and hSS were treated with vehicle, buprenorphine, or oxycodone for 10 days. On day 60, spheroids were homogenized and RNA was prepared using the Qiagen RNEasy Plus Micro Kit according to manufacturer instructions. RNA concentration and quality were determined using a NanoDrop, then total RNA was converted to cDNA using the Applied Biosystems High Capacity Reverse Transcription Kit. Real-time quantification of diluted cDNA was performed in triplicate reactions containing sample (4 ng), IDT Primetime Gene Expression Master Mix (2X), and IDT Primetime qPCR Assay (20X) on a BioRad CFX384 Real Time System. Cycling Conditions consisted of one cycle at 95°C for 3 min, followed by 40 cycles of denaturation (95°C for 15 s) and elongation (60°C for 1 min). The relative gene expression was calculated using the 2^–ΔΔCT^ method. The following IDT Primetime Gene Expression Assays were used: *DLX1* (Hs.PT.58.4632198), *DLX5* (Hs.PT.58.45646524), *LHX6* (Hs.PT.58.27682011), *ARX* (Hs.PT.58.213.7622), *NKX2.1* (Hs.PT.58.2461055), *ADCY3* (Hs.PT.569.2298693), *BCL11B* (Hs.PT.58.27217530), *MKI67* (Hs.PT.58.27920212), *PAX6* (Hs.PT.58.25914558), *SATB2* (Hs.PT.58.24560574), *TBR1* (Hs.PT.58.24815929), *TUBB3* (Hs.PT.58.20385221) and *GAPDH* (Hs.PT.39a.22214836).

### cAMP Assay

NOP receptor-mediated inhibition of adenylyl cyclase activity was determined by measuring cAMP accumulation in the presence of the adenylyl cyclase activator, forskolin, and the phosphodiesterase inhibitor, rolipram as previously described (Berg et al., 2007). Briefly, spheroids were incubated in wash buffer (HBSS containing 20 mM HEPES, pH 7.55) for 15 min at 37°C. Rolipram (100 μM, Sigma) was added along with the following drugs: buprenorphine (5 nM), nociceptin (150 nM, Tocris), or oxycodone (60 nM) in the presence or absence of the NOP Receptor antagonist, UFP-101 (10 μM, Tocris). Forskolin (10 μM, Sigma) was then added and spheroids were incubated for an additional 15 minutes. Incubation was terminated by aspiration of the wash buffer and addition of 500 μl of ice cold ethanol. The ethanol extracts from individual spheroids were dried under a gentle airstream and reconstituted in 100 μl of sodium acetate (pH 6.2) and the cAMP content was determined by radioimmunoassay (RIA).

### Multi-Electrode Array Assay

To record network activity, the Harvard MCS Multiwell-MEA-system, which contains a 12-electrode grid in each well of a 24 well plate, was used. Twenty-four hours before the MEA was conducted, spheroids were transferred to the MEA plate containing BrainPhys media (STEMCELL Technologies). For acute experiments, a 10-min baseline recording was followed by treatment with buprenorphine, or oxycodone in the presence of vehicle or the NOP receptor antagonist, UFP-101. Fifteen minutes later, 3 10-min recordings were performed. For the chronic experiments, hCS or fused hCS-hSS spheriods were treated with vehicle, buprenorphine, or oxycodone for 10 days. On day 60 or day 120, spheroids were transferred to MEA plates containing BrainPhys media and 3 10-min recordings were performed. Data was sampled at 20 kHz, digitized and analyzed using the Harvard Multiwell-Analyzer software with a 100 Hz high pass and 3.5 kHz low pass filter and an adaptive spike detection threshold was set at 8 times the standard deviation for each electrode. The results from each 10-min recording were averaged.

### Interneuron Migration

On day 30, hSS were infected with Lenti hDlx1/2b:GFP (gift from Dr. John Rubenstein). On day 40, one hSS and one hCS were transferred to an Eppendorf tube containing neural media and incubated without disruption for 3-4 days. After fusion was complete, fused spheroids were treated with buprenorphine or oxycodone from days 50-60. On day 60, fused spheroids were fixed, sliced and imaged. The total number of GFP + cells and the number of GFP + cells that migrated into the hCS were counted.

### Rat Experiments

Pregnant female rats were injected intraperitoneally with 1 mg/kg buprenorphine or 10 mg/kg oxycodone on gestational days 11-21, a time point that encompasses both cortical and subcortical neurogenesis and neuronal migration (Schepanski et al., 2018). Male and female pups were weaned on postnatal day 21 and all experiments were performed in adult animals (after postnatal day 42). Animals were maintained in a temperature-controlled environment on a 14:10 h light-dark cycle and had access to food and water *ad libitum*. All procedures were consistent with NIH guidelines (NIH publications no. 80-23, revised 1978) and approved by the Institutional Animal Care and Use Committee of the University of Texas Health Science Center at San Antonio.

### Rat Immunohistochemistry

Adult rats were transcardially perfused with saline followed by 4% paraformaldehyde. Brains were post-fixed and cryopreserved for at least 24 hours. Coronal sections (50 μm) through the medial prefrontal cortex were blocked (5% normal goat serum), incubated with a mouse anti-Lhx6 primary antibody (Santa Cruz, 1:250), then washed and incubated with a goat anti-mouse Alexafluor488 secondary antibody (Invitrogen). Sections were mounted, coverslipped with Prolong Gold Antifade Reagent (Molecular Probes), then imaged using a Zeiss AxioObserver inverted microscope. To analyze the migration of interneurons into the cortex, 10 equidistant bins through the medial prefrontal cortex were defined to determine the distribution of cells across the cortical layers (Meechan et al., 2012). Lhx6 + cells were counted in at least 3 sections per animal by a blind experimenter.

### Human Fetal Brain Tissue Analysis

Fetal brain bulk RNA sequencing dataset was downloaded from BrainSpan: Atlas of the Developing Human Brain^1^. RPKM (reads per kilobase transcripts per million mapped reads) values for OPRL1 gene were mined and normalized for each sample using the following formula RPKM(gene)/(ΣRPKM(all genes in the sample)) X 10^6. Graphpad Prism was used to plot OPRL1 normalized counts from 8-37 weeks post conception and median values were used to draw conclusions about the temporal expression pattern.

### Statistical Analysis

In all figures, data are shown as mean + S.E.M and n is indicated in the figure legend. Data was analyzed by one- or two-way ANOVA and the Holm-Sidak *post hoc* test was used when significant interactions were present. When comparing groups with unequal variances the non-parametric Kruskal-Wallis test was used followed by Dunn’s multiple comparison test. All tests were two-tailed, and significance was determined at *p* < 0.05. Effect size was calculated using: https://www.psychometrica.de/effect_size.html.

## Results

### Generation of Cortical and Subpallial Spheroids

To study the effect of buprenorphine during human brain development, we first generated cortical spheroids (hCS) and subpallial spheroids (hSS) from hiPSCs and ESCs using the method described by Pasca and colleagues Supplementary Figure 2A (PaŞca et al., 2015; Birey et al., 2017). Both cell lines expressed pluripotent stem cell markers (Supplementary Figure 1A) and maintained normal karyotype (Supplementary Figure 1B). RNA sequencing was used to confirm spheroid identity. Neither cortical spheroids nor subpallial spheroids expressed *NANOG*, a homeobox protein involved in maintaining embryonic stem cell pluripotency (Supplementary Figure 2B) (Chambers et al., 2007). Cortical spheroids expressed *PAX6*, a marker for dorsal forebrain progenitor cells (Georgala et al., 2011), and *NEUROD6*, a gene expressed ubiquitously throughout the fetal cerebral cortex (Supplementary Figure 2B) (Camp et al., 2015). These genes were not expressed in subpallial spheroids. Conversely, subpallial spheroids expressed *ARX*, *DLX2*, and *NKX2-1* (Supplementary Figure 2B). DLX2 has been shown to bind to *ARX* and regulate the migration of GABAergic neurons (Colasante et al., 2008). Similarly, *NKX2-1* is a homeobox transcription factor that regulates the fate specification and migration of GABAergic interneurons (Du et al., 2008). These subpallial markers were not observed in hCSs. Immunohistochemistry was used to demonstrate that hCS showed VZ-like structures and PAX6 expression (Supplementary Figure 2C). Cortical spheroids did not express ARX (Supplementary Figure 2C). Conversely, hSS presented both NKX2.1 + and ARX + cells (Supplementary Figure 2D). These data showed hCS and hSS resembled features of cortical and subpallial brain development *in vitro*.

### Buprenorphine Signals Through the Nociceptin Opioid Peptide Receptor in Cortical Spheroids

**FIGURE 1:**
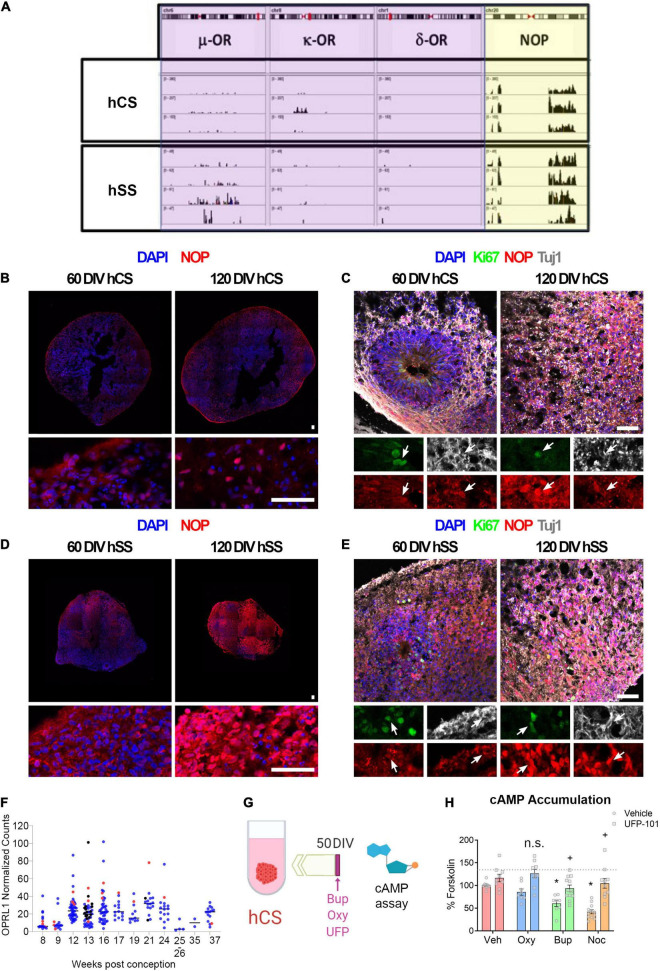
Buprenorphine signals through the nociceptin receptor. **(A)** RNA Sequencing found that both cortical and subpallial spheroids express the NOP receptor but not the μ-, κ - or δ-opioid receptors. *n* = 3-4. NOP expression was observed in both cortical **(B)** and subpallial **(D)** spheroids. NOP expression co-localized with both Ki67 and Tuj1 **(C,E)**. **(F)** Data from fetal brain tissue demonstrating NOP receptor expression in cortical and subpallial regions beginning at 12 weeks. Red dots indicate ganglionic eminences and striatum; blue and black dots indicate cortical tissue from two different samples. n = 1-2. The experimental design for cAMP assays is depicted in **(G)**. Buprenorphine, like the endogenous NOP receptor ligand, nociceptin, decreases cAMP accumulation. This effect is blocked by treatment with the NOP receptor antagonist, UFP-101 **(H)**. *n* = 8-12 organoids. Scale bar = 50 μm. n.s.,no significant: * and + p < 0.05. DIV,days *in vitro*.

Buprenorphine is thought to have its therapeutic effect via the μ- and κ-opioid receptors; however, buprenorphine also acts as a full agonist at the nociceptin opioid peptide (NOP) receptor (Kumar et al., 2014). Therefore, expression of these opioid receptors was first determined in hCS and hSS using RNA sequencing. In line with the pattern of opioid receptor expression in the human fetal brain (Allen Institute for Brain Science, 2008), neither the μ-, κ-, nor δ- opioid receptors were significantly expressed in either the cortical or subpallial spheroids (Figure 1A). However, the NOP receptor is expressed in both cortical and subpallial spheroids (Figure 1A). To identify the cell types expressing NOP, immunohistochemistry was used (Figures 1B-E). Robust NOP expression was observed in both cortical (Figure 1B) and subpallial (Figure 1D) spheroids at both 60 and 120 DIV. Further, NOP expression co-localized with Ki67 and Tuj1, suggesting this receptor is expressed on both proliferating cells and immature neurons (Figures 1C,E). In addition, we confirmed the expression of NOP receptor in human fetal tissue using publicly available dataset downloaded from BrainSpan, Atlas of the Developing Human Brain (See Text Footnote 1) (Figure 1F). After confirming NOP receptor expression, cAMP assays (Figures 1G-H) and multielectrode arrays (Supplementary Figures 3A-C) were performed on Day 50 cortical spheroids to determine if buprenorphine can have functional effects on the spheroids via the NOP receptor. Activation of the NOP receptor, which typically signals through Gi proteins, leads to an inhibition of adenylyl cyclase activity and decreases in cellular cAMP levels. Therefore, cAMP assays were first used to determine whether buprenorphine can activate intracellular signaling pathways via the NOP receptor. We found that buprenorphine decreased cAMP accumulation compared to vehicle-treated spheroids (Figure 1H, Two-way ANOVA: Interaction F(3,68) = 3.33, *p* < 0.05; Opioid F(3,68) = 11.90, *p* < 0.05; NOP Antagonist F(1,68) = 50.75, *p* < 0.05; Holm-Sidak *Post Hoc* Test: Vehicle-Vehicle vs. Buprenorphine-Vehicle *t* = 3.42, *p* < 0.05). These results are in line with the ability of nociceptin, the endogenous NOP receptor ligand, to decrease cAMP accumulation (Figure 1H, Holm-Sidak *Post Hoc* Test: Vehicle-Vehicle vs. Nociceptin-Vehicle *t* = 5.48, *p* < 0.05). Pretreatment with the NOP receptor antagonist UFP-101 completely reversed buprenorphine- and nociceptin-induced decrease in cAMP accumulation (Figure 1H, Buprenorphine-Vehicle vs. Buprenorphine-UFP *t* = 3.16, *p* < 0.05, Nociceptin-Veh vs. Nociceptin UFP *t* = 6.32, *p* < 0.05), confirming that in hCS, buprenorphine can signal through the NOP receptor.

To determine whether buprenorphine’s inhibition of cAMP signaling was accompanied by a change in neural activity, MEA recordings of cortical spheroids were then conducted to investigate changes in extracellular activity. Following acute exposure to the opioids, there was no significant effect of either oxycodone or buprenorphine on neuronal activity (Supplementary Figures 3A-C). To then determine if chronic opioid exposure affects cortical development, hCS were treated with buprenorphine or oxycodone for 10 days (day 50-60) and qPCR and MEAs were performed (Supplementary Figures 3D-G). While chronic treatment with either buprenorphine and oxycodone altered expression of some genes in hCS (Supplementary Figure 3E), neither drug produced significant changes in cortical activity (Supplementary Figures 3F-G). Similarly, when hCS were treated chronically with opioids (day 50-60), then tested at day 120, there were also no significant differences in neuronal activity (Supplementary Figures 3H-J).

### Buprenorphine Alters Markers of Interneuron Development in Subpallial Spheroids

**FIGURE 2:**
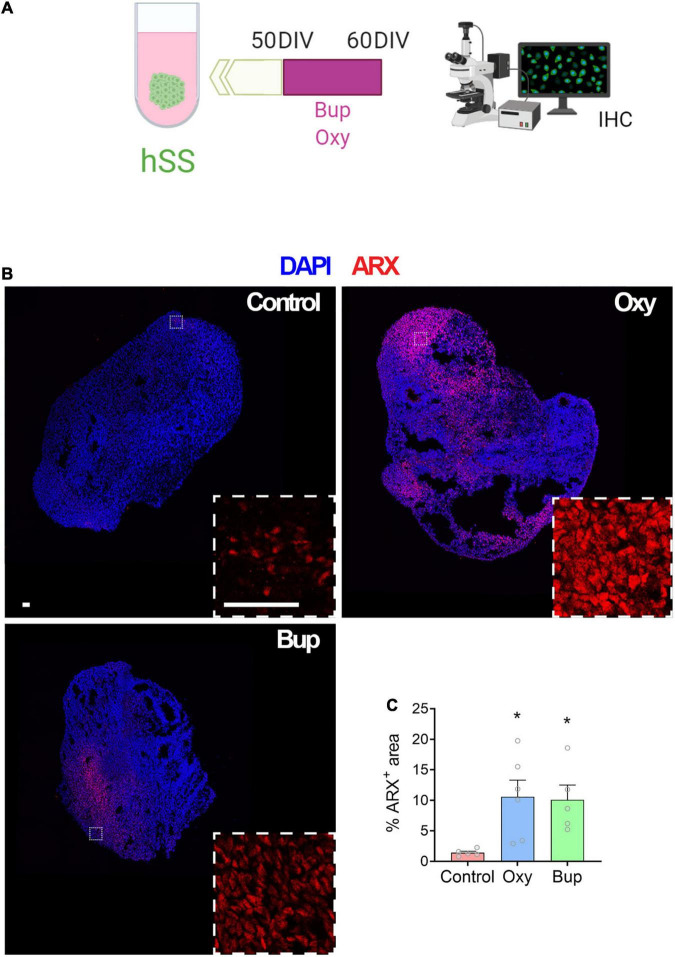
Buprenorphine alters markers of interneuron development in subpallial spheroids. The experimental design is depicted in **(A).** Representative images of ARX immunolabeling **(B)**. Both oxycodone and buprenorphine treatment increase ARX area in subpallial spheroids **(C)**. *n* = 5-6 organoids. Scale bar = 50 μm. *p < 0.05. DIV,days *in vitro*; IHC,Immunohistochemistry.

After demonstrating that buprenorphine can influence the intracellular signaling in cortical spheroids via the NOP receptor, the effect of buprenorphine on subpallial spheroids was also determined. Day 60 subpallial spheroids were analyzed by qPCR (Supplementary Figures 4A) and immunohistochemistry (Figure 2A) after a 10-day treatment with oxycodone or buprenorphine. First, qPCR was used to measure mRNA expression of *ARX*, *DLX1*, *LHX6*, and *NKX2-1*, genes involved in the development and migration of interneurons (Wonders and Anderson, 2005). In contrast to oxycodone, buprenorphine slightly increased interneuron gene expression although this was not statistically significant (Supplementary Figure 4B; Two-way ANOVA: Opioid F (2,164) = 3.06, *p* < 0.05). Then, ARX immunolabeling was performed and a significant increase in ARX + cells in hSS after opioid exposure was observed (Figures 2B,C). Moreover, no difference was found in the number of AC3 + cells (cleaved caspase 3) after opioid treatment (data not shown). These data suggest that opioids can affect interneuron progenitors during brain development without affecting cell survival.

### Buprenorphine Alters Interneuron Migration and Disrupts Network Activity in Fused Spheroids

**FIGURE 3:**
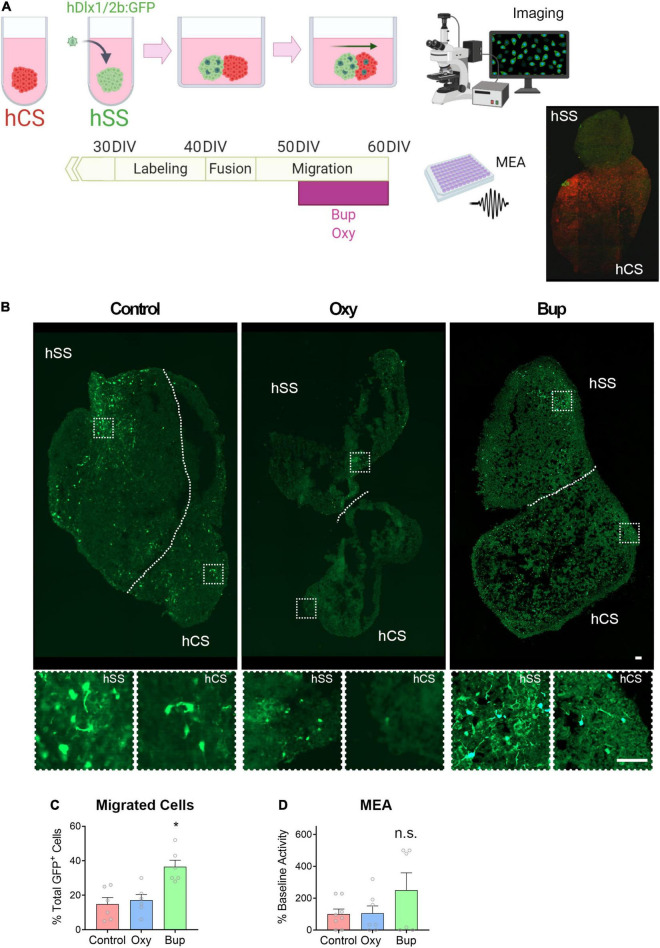
Buprenorphine alters interneuron migration into cortical spheroids. The experimental design is depicted in **(A)**. Representative images of fused spheroids are shown in **(B)**. Buprenorphine increased the number of GFP + cells that migrated into the cortical spheroid compared to control- and oxycodone-treated spheroids, n = 6 organoids **(C)**. Buprenorphine exposure produces a trend toward increased network activity compared to control- and oxycodone-treated fused spheroids, *n* = 6-7 organoids **(D)**. Scale bar = 50 μm. * *p* < 0.05, n.s., no significant: DIV,days *in vitro.*

Migration of interneurons from the ganglionic eminences into the cortex is essential to cortical development (Wonders and Anderson, 2005). In order to determine the effect of opioids on migrating interneurons, subpallial and cortical spheroids were fused and the migrated GFP-labeled interneurons was analyzed after 20 days (Figure 3A). There was a significant effect of opioid treatment on the percent of GFP-positive cells that migrated into the cortical spheroids from the subpallial area (Figures 3B,C; One-way ANOVA F = 10.63, *p* < 0.05). *Post hoc* analysis demonstrated that buprenorphine produced a significant increase in the percentage of GFP-positive cells that migrated into the cortex compared to both control- and oxycodone-treated spheroids (Holm-Sidak: Control vs. Buprenorphine *t* = 4.201; Buprenorphine vs. Oxycodone *t* = 3.748). Next, we determined if this change in interneuron migration had an effect on network activity using MEA (Figure 3D). Cortical spheroids were treated with vehicle, buprenorphine, or oxycodone for 10 days. At day 60, MEAs were conducted to determine the effect of chronic opioid exposure on the extracellular activity of the spheroids. Although not significant, buprenorphine-treated spheroids exhibited a trend toward increased activity compared to the vehicle- and oxycodone-treated spheroids [Figure 3D, One-way ANOVA F(2,11) = 0.89, *p* > 0.05; Effect size: control vs. oxy = 0.049 (no effect), control vs. bup = 0.806 (large effect)].

### Prenatal Buprenorphine Exposure Alters Interneuron Migration in the Rodent Cortex

**FIGURE 4:**
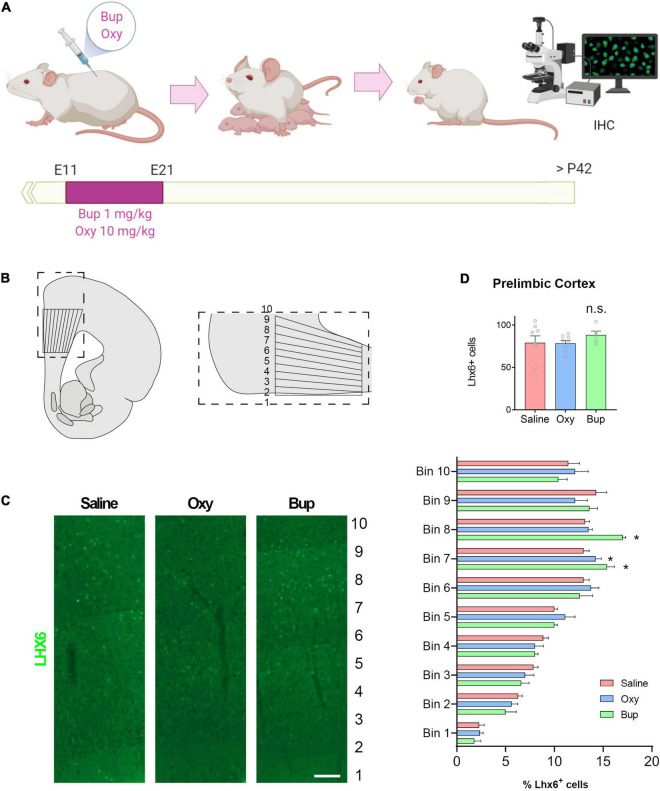
Prenatal buprenorphine alters interneuron migration in the rodent cortex. The experimental design is depicted in **(A)**. The region of the medial prefrontal cortex analyzed is shown in **(B)**. Representative images of Lhx6 + cells are shown in **(C)**. The total number of Lhx6 + cells in the prelimbic region (PrL) of the mPFC is not altered by *in utero* opioid exposure. The distribution of Lhx6 + cells throughout the PrL is altered by buprenorphine exposure **(D)**. *n* = 5-8 rats from 3 litters per group. Scale bar = 100 μm. *p < 0.05, n.s.,no significant: IHC,Immunohistochemistry.

To determine if buprenorphine can also disrupt interneuron migration *in vivo*, pregnant rats were injected with buprenorphine or oxycodone on gestational days 11-21 (Figure 4A). In adult offspring, the total number and distribution of Lhx6 + interneurons were analyzed throughout the medial prefrontal cortex (mPFC; Figure 4B). Lhx6, a LIM homeodomain transcription factor, is involved in the development of specific classes of GABAergic interneurons, namely somatostatin and parvalbumin subtypes, and expression is maintained in mature interneurons (Zhou et al., 2015).Prenatal opioid exposure had no effect on the total number of Lhx6 + positive cells in the mPFC (Figure 4D; One-way ANOVA F = 0.65, *p* > 0.05) or the thickness of the cortex (data not shown). However, the laminar distribution of Lhx6 + cells was altered by prenatal buprenorphine exposure (Figures 4C,D; Mixed effects ANOVA Layer F(2.9,20.4) = 67.45, *p* < 0.05). Specifically, in bins 7 and 8, there were significantly more Lhx6 + cells in the buprenorphine-treated animals (Holm-Sidak Bin 7 *t* = −2.4, *p* < 0.05; Bin 8 *t* = −3.9, *p* < 0.05). Together, these results suggest that prenatal buprenorphine exposure can alter interneuron migration into the developing cortex, and the altered distribution of interneurons can persist into adulthood.

## Discussion

In the current experiments, we used data from fetal brain tissue, human brain spheroids, and a rodent model to provide preliminary evidence that prenatal buprenorphine exposure can alter interneuron development and migration and disrupt cortical network activity via the nociception opioid peptide (NOP) receptor. Buprenorphine, the preferred treatment for opioid use disorder (OUD) in pregnant women (ACOG Committee on Health Care for Underserved Women and American Society of Addiction Medicine, 2012), acts as a partial agonist at μ− and κ-opioid receptors to alleviate withdrawal symptoms while blocking the effect of exogenously administered opioids (Kumar et al., 2014). However, this drug is also a full agonist at the NOP receptor (Kumar et al., 2014). The NOP receptor, encoded by the *OPRL1* gene, is a member of the opioid family of G protein-coupled receptors but is not activated by classical opioids with known abuse liability (Zaveri, 2016). Upon ligand binding, the NOP receptor acts through Gα_i/o_ to inhibit adenylate cyclase, activate mitogen-activated protein kinases, increase K^+^ conductance, and inhibit Ca^2+^ conductance (Mogil and Pasternak, 2001; New and Wong, 2002). Previous work has demonstrated that the NOP receptor is expressed throughout the developing rat and human brain, including in the neocortex (Neal et al., 2001). We confirmed these findings using a published RNA sequencing data set from human fetal brain tissue. Specifically, we found that the NOP receptor is expressed in cortical and subpallial fetal brain tissue as early as 12 weeks post-conception and remains elevated until birth, suggesting that buprenorphine treatment *in utero* has the potential to affect cortical development through its activity at the NOP receptor.

After demonstrating that the NOP receptor is present in fetal brain tissue, we then moved to an *in vitro* model to confirm that NOP receptor is expressed in hCS and hSS; and buprenorphine can signal through this receptor in developing cortical tissue. Specifically, we used hPSCs to grow cortical spheroids, which resemble the developing cortex with ventricular zone-like structures and progenitor cells expressing *PAX6*. We used RNA sequencing to demonstrate that the NOP receptor is present in cortical and subpallium spheroids and its expression co-localized with Ki67 + cells (proliferative cells) and Tuj1 + (neurons). Interestingly, we did not observe expression of the μ-, k-, or d- opioid receptors in the human cortex, which is in line with results from fetal brain suggesting that these receptors are more prominent in subcortical regions and not expressed until later in development (Allen Institute for Brain Science, 2008). Then, we used cAMP and MEA assays in these cortical spheroids to demonstrate that the NOP receptor is not only present but that buprenorphine can signal via this receptor in developing brain tissue.

The cortex is made up of a variety of cell types, including interneurons, which are derived from a group of subpallial brain regions, the ganglionic eminences, before migrating tangentially into the cortex (Wonders and Anderson, 2005). Therefore, we also used hPSCs to grow subpallial spheroids, which contain GABA-expressing neurons and express genes associated with interneuron development (*NKX2-1*, *ARX*, and *DLX2*). We found that chronic buprenorphine exposure increased markers of interneuron progenitors in subpallial spheroids. Further, when cortical and subcortical spheroids were fused (Bagley et al., 2017), we found that buprenorphine exposure altered interneuron migration, leading to an increase in interneurons in cortical spheroids. This increase in interneuron migration was accompanied by a trend toward an increase in network activity in fused spheroids exposed to buprenorphine chronically. These results may seem contradictory as interneurons signal via the inhibitory neurotransmitter, GABA. However, high intracellular chloride concentrations in the developing brain can result in depolarization of the cell when GABA binds to its ionotropic receptors (Ben-Ari et al., 1989; Owens et al., 1996). Interestingly, the excitatory action of GABA in the developing brain has been shown to induce synaptogenesis (Wolf et al., 1986) and produce long-term changes in synaptic efficacy (Caillard et al., 1999a,b), suggesting that alterations in the development of GABAergic interneurons by buprenorphine may shape the development of the cortex.

Network activity in the adult cortex is regulated by a complex interplay between excitatory and inhibitory neurons. Excitatory pyramidal cells release glutamate and are primarily responsible for transmitting information between brain regions. Conversely, inhibitory interneurons use GABA to signal and act locally to maintain control over individual pyramidal cells and to regulate oscillations across neuronal assemblies (Marin, 2012). After demonstrating that buprenorphine can alter interneuron migration in the developing cortex, we next used a rodent model of prenatal opioid exposure to show that buprenorphine exposure *in utero* produces changes in interneuron distribution throughout the adult cortex. These findings may be significant as interneuron dysfunction has been associated with a variety of neurodevelopmental and psychiatric disorders, including schizophrenia, autism, and intellectual disability (Marin, 2012). For example, schizophrenia patients have decreased expression of the GABA synthesizing enzyme, glutamic acid decarboxylase (GAD), in the prefrontal cortex, an effect that seems to reflect not a loss of cells but rather a loss of function in specific subclasses of interneurons (Donegan and Lodge, 2017). We have recently demonstrated that disrupting interneuron function in the cortex can produce schizophrenia-like deficits in social interaction and cognitive function in otherwise healthy animals (Perez et al., 2019). Further, our lab and others have shown that restoring interneuron function in the prefrontal cortex in a rodent developmental disruption model can attenuate behavioral deficits (Donegan et al., 2018). Together with the current findings, these results suggest buprenorphine alters the development of cortical circuits implicated in the pathology of neurodevelopmental and psychiatric disorders.

Interestingly, the NOP receptor and its endogenous ligand, nociceptin, have also been implicated in psychiatric disease (Post et al., 2016; Khan et al., 2018). In adults, the receptor is highly expressed in brain regions associated with mental illness, including the cortex, hippocampus, amygdala, thalamus, hypothalamus, and dorsal raphe (Neal et al., 2001; Berthele et al., 2003; Lambert, 2008; Kimura et al., 2011; Lohith et al., 2012). Further, nociceptin levels are elevated in patients suffering from bipolar disorder and major depression (Wang et al., 2009). A recent proof of concept clinical trial demonstrated that the NOP receptor antagonist, LY2940094, improved depression scores compared to placebo (Post et al., 2016). These limited human findings are corroborated by animal models. Specifically, antidepressant-like effects in the forced swim test have been observed in both NOP receptor knock-out animals (Rizzi et al., 2011) and in wild-type rodents treated with a NOP receptor antagonist (Gavioli and Calo, 2013; Post et al., 2016). Activation of the NOP receptor has also been shown to impair working memory, one cognitive function associated with schizophrenia (Goeldner et al., 2008). While the current experiments did not examine how buprenorphine exposure *in utero* may affect NOP receptor function in adulthood, previous work has demonstrated that in rodents, prenatal buprenorphine exposure can decrease G protein coupling to NOP receptors (Hou et al., 2004). Future experiments will be required to determine if buprenorphine activation of the NOP receptor during development can affect adult NOP receptor function and lead to the development of psychiatric disorders.

The current results suggesting that buprenorphine can alter cortical development are in line with human studies that demonstrate that buprenorphine exposure *in utero* may have long-term developmental consequences. Specifically, 5-6 year-old children exposed to buprenorphine *in utero* have been shown to have deficits in motor skills and memory. These children also had increased hyperactivity, impulsivity, and attention problems compared to peers matched for neonatal abstinence syndrome, gender, and socio-economic factors (Sundelin Wahlsten and Sarman, 2013). Other studies, however, have failed to demonstrate that prenatal buprenorphine exposure produces deficits in cognitive development, language abilities, sensory processing, and temperament over the first 3 years of life (Kaltenbach et al., 2018). These contradictory findings in humans may be a result of the numerous potential confounding factors at play, including the severity of neonatal abstinence syndrome and obstetric complications (e.g., low birth weight, etc.) as well as the home environment. Further, these studies are limited to infants and young children. However, the current studies suggest that buprenorphine exposure *in utero* may influence circuits involved in psychiatric disorders, which often do not present until adulthood. There are currently no human studies that follow buprenorphine exposed babies into adulthood. However, rodent studies show that prenatal buprenorphine exposure can decrease neurogenesis and increases depression- and anxiety-like behavior in adult animals (Wu et al., 2014; Gholami et al., 2020), suggesting that buprenorphine may have long-term consequences.

In conclusion, a large body of research indicates that OUD can have severe and immediate consequences on a fetus, including low birth weight, sudden infant death, respiratory complications and Neonatal Abstinence Syndrome (Tobon et al., 2019). Buprenorphine has been shown to improve outcomes in infants, decreasing the incidence, severity, and duration of Neonatal Abstinence Syndrome compared to mothers using illicit opioids (Binder and Vavrinková, 2008) and those that receive other treatments for OUD (Brogly et al., 2014; Zedler et al., 2016). Therefore, our results should not be interpreted as a reason to stop the use of buprenorphine in pregnant women with OUD. However, our findings suggest that more research should be done to increase our understanding of the long-term consequences of buprenorphine exposure *in utero*, and to find novel therapeutics, particularly those that do not target the NOP receptor.

## Data Availability Statement

The datasets presented in this study can be found in online repositories. The names of the repository/repositories and accession number(s) can be found below: NCBI (accession: PRJNA818652).

## Ethics Statement

The studies involving human participants were reviewed and approved by University of Texas at San Antonio, San Antonio, TX, United States. The patients/participants provided their written informed consent to participate in this study. The animal study was reviewed and approved by University of Texas Health Science Center, San Antonio, TX, United States.

## Author Contributions

VN-E and JD: design, collection and assembly of data, data analysis and interpretation, and manuscript writing. CM, HR, TC, and PV: collection and assembly of the data. KB: conception and design, data analysis and interpretation. DL and JH: conception and design, data analysis and interpretation, financial support, manuscript editing, and final approval of manuscript. All authors contributed to the article and approved the submitted version.

## Conflict of Interest

The authors declare that the research was conducted in the absence of any commercial or financial relationships that could be construed as a potential conflict of interest.

## Publisher’s Note

All claims expressed in this article are solely those of the authors and do not necessarily represent those of their affiliated organizations, or those of the publisher, the editors and the reviewers. Any product that may be evaluated in this article, or claim that may be made by its manufacturer, is not guaranteed or endorsed by the publisher.

## Footnotes

1
https://brainspan.org/
