# Supplementary figures and images for: Buprenorphine Exposure Alters the Development and Migration of Interneurons in the Cortex
Source: Front Mol Neurosci. 2022 May 4;15:889922. doi: 10.3389/fnmol.2022.889922 (PMC9115473; doi:10.3389/fnmol.2022.889922)

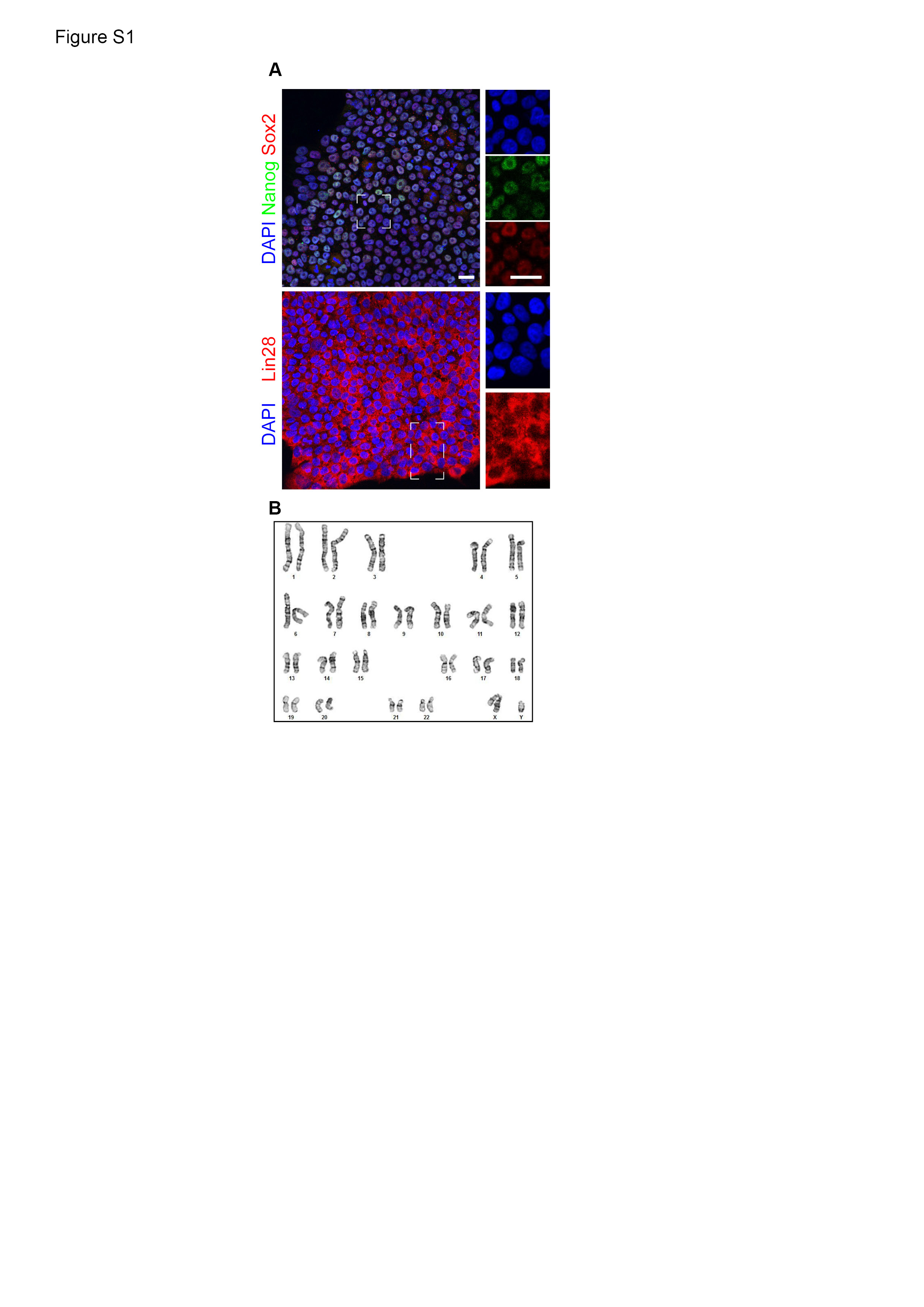

Supplement: Supplementary file 1 [file Image_1.JPEG]

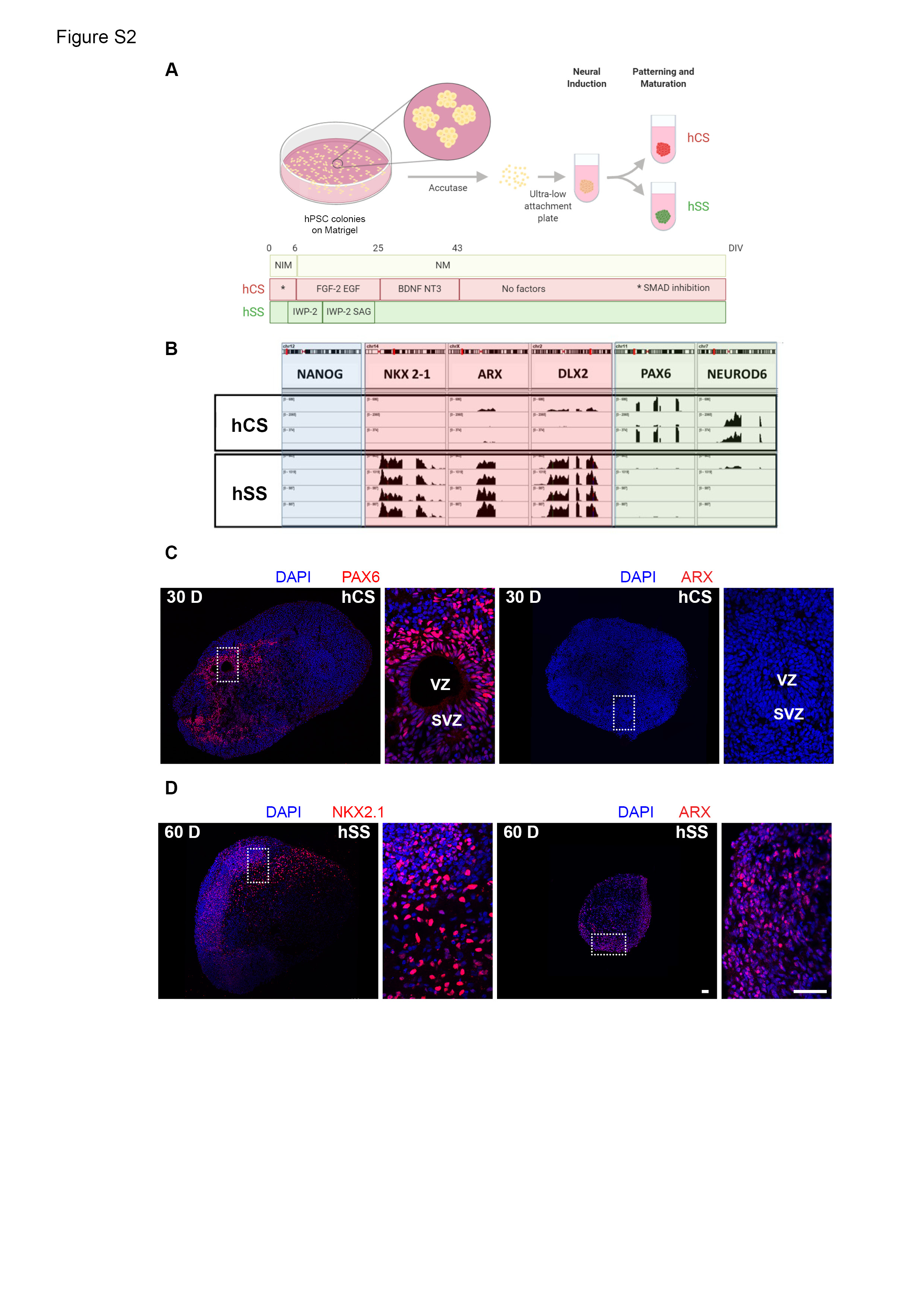

Supplement: Supplementary file 2 [file Image_2.JPEG]

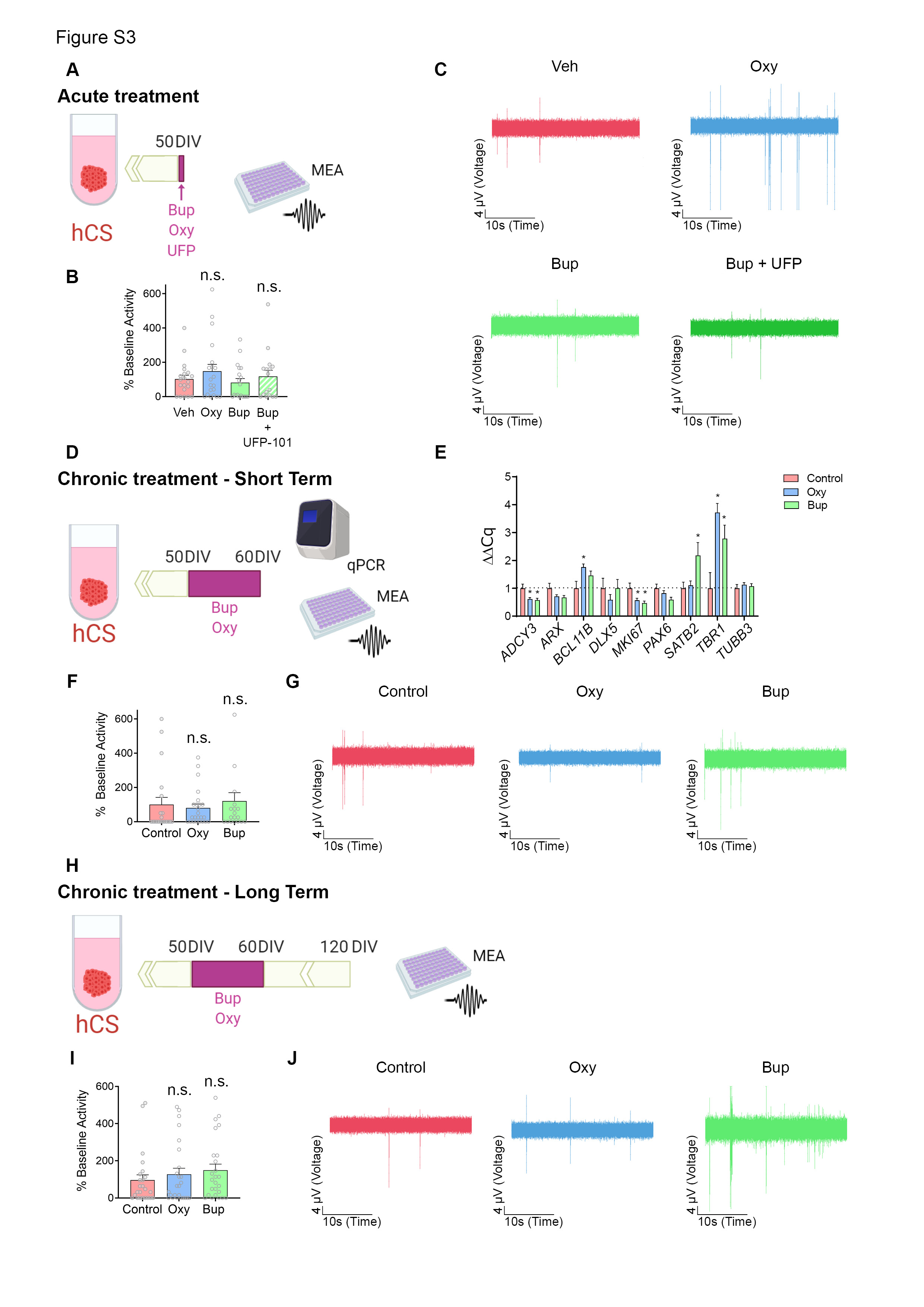

Supplement: Supplementary file 3 [file Image_3.JPEG]

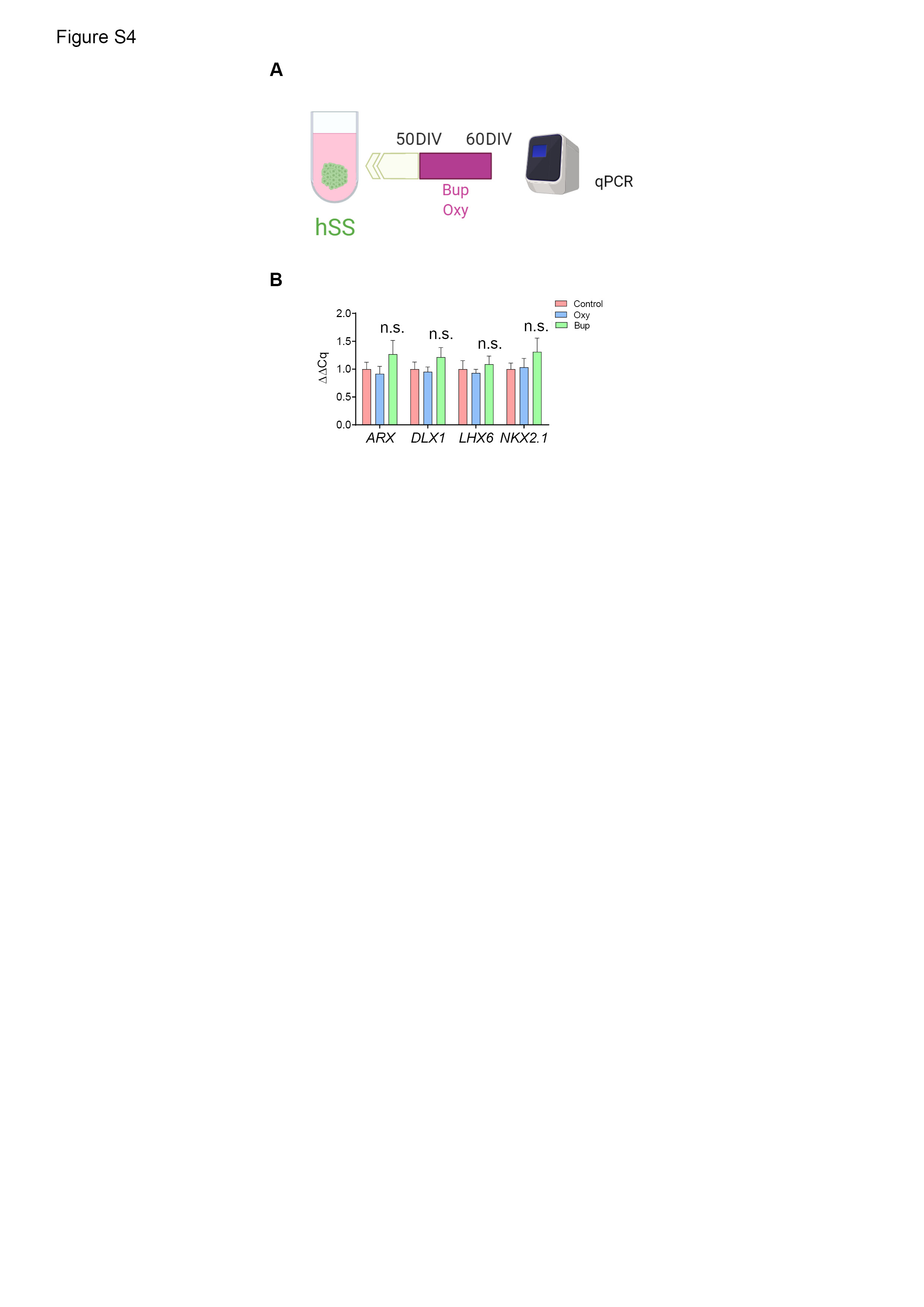

Supplement: Supplementary file 4 [file Image_4.JPEG]
